# HPMA Copolymer-Based Nanomedicines in Controlled Drug Delivery

**DOI:** 10.3390/jpm11020115

**Published:** 2021-02-10

**Authors:** Petr Chytil, Libor Kostka, Tomáš Etrych

**Affiliations:** Institute of Macromolecular Chemistry, Czech Academy of Sciences, Heyrovsky Sq. 2, 162 06 Prague, Czech Republic; chytil@imc.cas.cz (P.C.); kostka@imc.cas.cz (L.K.)

**Keywords:** HPMA copolymers, EPR effect, drug delivery, controlled release, nanomedicines

## Abstract

Recently, numerous polymer materials have been employed as drug carrier systems in medicinal research, and their detailed properties have been thoroughly evaluated. Water-soluble polymer carriers play a significant role between these studied polymer systems as they are advantageously applied as carriers of low-molecular-weight drugs and compounds, e.g., cytostatic agents, anti-inflammatory drugs, antimicrobial molecules, or multidrug resistance inhibitors. Covalent attachment of carried molecules using a biodegradable spacer is strongly preferred, as such design ensures the controlled release of the drug in the place of a desired pharmacological effect in a reasonable time-dependent manner. Importantly, the synthetic polymer biomaterials based on *N*-(2-hydroxypropyl) methacrylamide (HPMA) copolymers are recognized drug carriers with unique properties that nominate them among the most serious nanomedicines candidates for human clinical trials. This review focuses on advances in the development of HPMA copolymer-based nanomedicines within the passive and active targeting into the place of desired pharmacological effect, tumors, inflammation or bacterial infection sites. Specifically, this review highlights the safety issues of HPMA polymer-based drug carriers concerning the structure of nanomedicines. The main impact consists of the improvement of targeting ability, especially concerning the enhanced and permeability retention (EPR) effect.

## 1. Introduction

Generally, polymer nanomedicines are macromolecule-based water-soluble, particular or micellar constructs with the 1–100 nm size range in at least one dimension that can load or attach active molecules, e.g., drugs, to a carrier to enable targeted delivery and/or site-specific controlled release of biologically active molecules [1]. Polymer nanomedicines are generally employed for the delivery of a variety of drugs, but their most important research applications fall in the field of anti-inflammatory, antibiotics, and mainly anticancer drug delivery [2,3]. Nanomedicines delivering antibiotics, anti-inflammatory, or anticancer drugs substantially reduce the overall toxicity of carried chemotherapeutics, accumulate in the inflamed or solid tumor tissue, and highly improve drug solubility, stability against degradation and biotransformation, and pharmacokinetics [4,5]. “Impeccable” nanomedicines deliver drugs directly into the target cells and their compartments with minimal drug release in the bloodstream, and thus reducing side effects on healthy tissue.

Polymer-based nanomedicines are intensively studied for several decades, and the concept of polymer-drug constructs became generally accepted and thoroughly studied [6]. Polymer nanomedicines restrain much of the drawbacks associated with the application of conventional low-molecular-weight chemotherapeutics, such as short circulation time, a low area under the curve, and significant systemic toxicity. Moreover, polymer-based nanomedicines enable targeted delivery and controlled drug release in the treated tissue. Although the application of polymer-based nanomedicines is wide, in this review, we focus mainly only on polymer-drug conjugates intended for cancer treatment with the possible application also to anti-inflammatory compounds or antibiotics delivery. A potent anticancer efficacy devoid of substantial systemic toxicity has been thoroughly documented in tumor-targeted therapies based on conjugates containing various consisting of *N-*(2-hydroxypropyl) methacrylamide (HPMA) copolymers (pHPMAs). They are biocompatible, non-toxic, and non-immunogenic and enable the attachment of the drug to the carrier via suitable biodegradable spacer responsive to various tumor- or inflammation-site associated stimuli. Importantly, pHPMA or other water-soluble polymers are used in drug delivery due to their hydrophilic nature of the polymer backbone, which hydration, i.e., tightly bound water layer, increases the energetic barrier of protein or other biomacromolecules adsorption during the blood circulation [7,8]. Moreover, the binding of active compounds to the pHPMA can solubilize water-insoluble drugs, dramatically improve pharmacokinetics, and eliminate the side effects of drugs. Recently, many examples of polymer prodrugs showed prolonged blood clearance, enhanced localization in solid tumors via the enhanced and permeability retention (EPR) effect [9], followed by controlled release of the active drug in target tumor tissues or cells. For example, an excellent antitumor activity of polymer prodrugs containing cytostatics, e.g., doxorubicin (Dox), pirarubicin (THP), or docetaxel bound by pH-sensitive spacer stable during circulation in the blood (pH 7.4), but rapidly hydrolysable in tumors upon pH decrease to pH 6 in the tumor microenvironment or pH 5–5.5 in endosomes/lysosomes of target cancer cells, was shown repeatedly [10,11,12].

Polymer design, including polymer structure, molecular weight, spacer structure, etc., strongly influences the overall therapeutic activity. An enormous effort has been pushed on the development of pHPMA-based nanomedicines taking advantage of the EPR effect. Their high molecular weight (HMW) prevents fast blood clearance of carried drugs and thus enables their increased uptake in solid tumors. The extent of accumulated polymer carriers primarily depends on their molecular weight [13]. Nevertheless, there is an upper limit in molecular weights due to the slower extravasation of polymers with quite high *M*_w_. For example, the star-like pHPMA-Dox conjugate with *M*_w_ = 1,000,000 g/mol was accumulated much lower than the conjugate with *M*_w_ = 600,000 g/mol [14]. To prevent the undesired accumulation of carriers in the body, which can lead to serious long-term side-effects, the elimination of carriers after fulfilling their role must be ensured.

The polymer biomaterial serving as the carrier should be removed from the body after the carried cargo is delivered to the target tissue and released. The clearance of polymer carriers by glomerular filtration is mainly controlled by the size of the polymer coil in solution, which is correlated to polymer *M*_w_. Non-charged copolymers with a size smaller than the size of glomerular pores can permeate through them, resulting in the elimination of polymers from the body by urine. Polymer carriers with biodegradable backbone, e.g., poly (glutamic acid) [15], can be hydrolyzed and degraded directly in the body. Nevertheless, methacrylamide-based polymer biomaterials are non-degradable in general; thus, their direct biodegradation cannot be taken into consideration. Polymer carriers consisted of non-degradable polymers that undergo renal filtration only up to a certain limit of *M*_w,_ which differs for various types of polymers. For example, for the vinylic type of polymers, it is known that this limit is about 50,000 g/mol [16]. Nevertheless, a thorough study using various water-soluble HPMA polymer-Dox conjugates showed that even linear polymers with *M*_w_ around 70,000 g/mol had been found in the urine [17]. Interestingly, star conjugates with *M*_w_ around 50,000 g/mol have been found in urine, nicely illustrating the role of flexibility or vice versa rigidity of polymer carriers. Here, more flexible linear polymer chains above 50,000 g/mol were able to penetrate through glomerular pores (although slower than that below this value), rather rigid branched star polymer structures could not.

Hence, HMW pHPMA carriers exhibiting the significant EPR effect should contain biodegradable linkages between single non-degradable polymer chains with *M*_w_ below the limit of renal filtration to increase passive targeting and to allow the following elimination of the carrier fragment from the body. Alternatively, HMW supramolecular structures such as micelles formed by self-assembly of amphiphilic copolymers with a molecular weight below the limit of the renal threshold have been proposed. The variety of such HMW polymer carriers is described in Chapter 3.

The EPR effect is a vascular issue that is dynamic and flexible. Vascular dilatation by various mediators or tumor-selective passive gap opening augment the EPR effect and thus enhance the accumulation of nanomedicines in tumor tissue. Such modulation of therapy using nanomedicines is discussed in Chapter 4. The effectiveness and applicability of nanomedicines designed for passive versus active tumor targeting are considered in Chapter 5. Nevertheless, prior demonstration of the variety of pHPMA carriers, let us focus on safety issues related to HPMA homopolymer in the following chapter.

## 2. Safety Features of HPMA Polymers Per Se

The pHPMAs have been “invited to the stage” of drug delivery in the early 1970s. In 1974 two patent applications were filed, which covered the synthesis of *N*-substituted (meth) acrylamides containing oligopeptide sequences and their application as drug and other biologically active compounds carriers [18,19]. The HPMA polymer was originally developed as a fully synthetic plasma expander under the commercial name Duxon™ [20,21,22]. Therefore, early in the 1980s, HPMA polymer was also tested in vitro as well as in vivo in several animal models [23,24,25,26,27,28] for biocompatibility and immunogenicity.

Selected types of cell lines have been used to evaluate the toxicity of Duxon™ (HeLa, L-cells, WI-38), and none of the tested cell lines showed any toxicity. Moreover, Duxon™ in saline solution was completely apirogenic, as was demonstrated in guinea pigs after intraperitoneal administration of 5 mL of the 5% solution of Duxon™ in saline [22,23]. As a further test of HPMA polymer biocompatibility, an attempt to heal experimental implants of pHPMA crosslinked with 1 mol% methylene-bis-acrylamide subcutaneously implanted in experimental rats and pigs was chosen. Macroscopically, the implant was well tolerated by the organism in all groups and at all time intervals, both in rats and pigs, and did not elicit any adverse reaction [29]. The implant was encapsulated with a fine fibrous sheath. Microscopically, in the first days after implantation, the implant was surrounded by a border of polynuclear leukocytes and fibrin. On the tenth day after implantation, in most cases, the polynuclear leukocytes disappear, and the implant was surrounded by a sheath of fine collagen fibers and fibrocytes. Importantly, the sheath was highly vascularized. At longer intervals, the collagen fibers became coarser, and the sheath was less cellular. The vascularization of the capsule persists. The histological picture does not change from the tenth day after implantation [25,30].

Šprincl et al. in 1976 observed that, in some organs (spleen, lymph nodes), the amount of the polymer first decreases and then increases again, which was attributed to the release and trapping of the polymer in RES. More pronounced accumulation was observed in organs with phagocytic activity. The usual histological examination did not reveal any changes in the individual organs [21]. Říhová et al. concluded that HPMA homopolymer is not recognized as a foreign macromolecule in any of the five inbred strains of mice, and no detectable antibodies were formed against it [31].

## 3. Structural Aspects

The preferable way of pHPMA carrier elimination is renal filtration; thus, the molecular weight of non-degradable polymer carriers or fragments remaining after biodegradation of HMW polymer carriers must be below the limit of renal filtration. The pHPMA carrier, which does not meet such criterium, could be excluded from the organism by a very slow process through the hepatobiliary pathway, as documented elsewhere [32,33]. However, this option is not ideal and generally preferred as the slow clearance of even biocompatible polymer carrier could cause in the long term in the body adverse effects unnoticed in the experiments focusing on the acute toxicity of the used polymer biomaterial.

This chapter is divided into three subchapters. The first chapter is focused on the employment of linear polymer with *M*_w_ reaching the limit of renal filtration. The influence of the synthetic method on the properties of pHPMA nanomedicines is shown in detail. The second chapter presents various HMW polymer constructs containing biodegradable linkages in their structure. Moreover, the third part introduces HMW supramolecular structures formed by self-assembly of amphiphilic pHPMAs.

### 3.1. Linear Polymer Carriers

The improvement of controlled polymerization techniques in the last two decades enabled the synthesis of polymer carriers with quite narrow dispersity. Specifically, the reversible addition–fragmentation transfer (RAFT) polymerization has been successfully employed for the synthesis of HPMA copolymers and their drug conjugates [34,35,36]. Recently, also Cu-RDRP polymerization (part of atom transfer radical polymerization (ATRP) technique family) was employed for successful HPMA polymerization and copolymerization. Here, the reaction was optimized with respect to monomer conversion (82–99%), product dispersity (<1.25), and *M*_w_ control (from 20,000 up to 100,000 g/mol). For this purpose, different chlorine-based initiators in conjunction with a CuCl/CuCl_2_/PMDETA catalytic system have been used. The utility of the optimized method was exemplified in the preparation of the pHPMA carrier having the anticancer drug Dox conjugated through a pH-sensitive hydrazone bond [37].

A preliminary study comparing pHPMA-drug conjugates bearing Dox bound by pH-sensitive hydrazone bond differing in the dispersity of polymer carriers showed the potential of low-dispersed conjugates prepared using RAFT polymerization [38]. While the polymer precursors have the same *M*_w,_ about 30,000 g/mol, the dispersity was highly different (*Ð* = 1.13, or 1.75) due to the utilization of controlled RAFT or free radical polymerization, respectively. The higher antitumor activity of the low-dispersed conjugate could be ascribed to an enhanced tumor accumulation due to the retention of polymer chains with sufficient molecular weights and vice versa the absence of fraction of polymer chains with lower molecular, which are fast cleared from the blood circulation and removed by urine. Therefore, it can be expected that such polymer–drug conjugates will be efficient in the treatment of solid tumors and still capable of carrier removal from the body.

**Figure 1:**
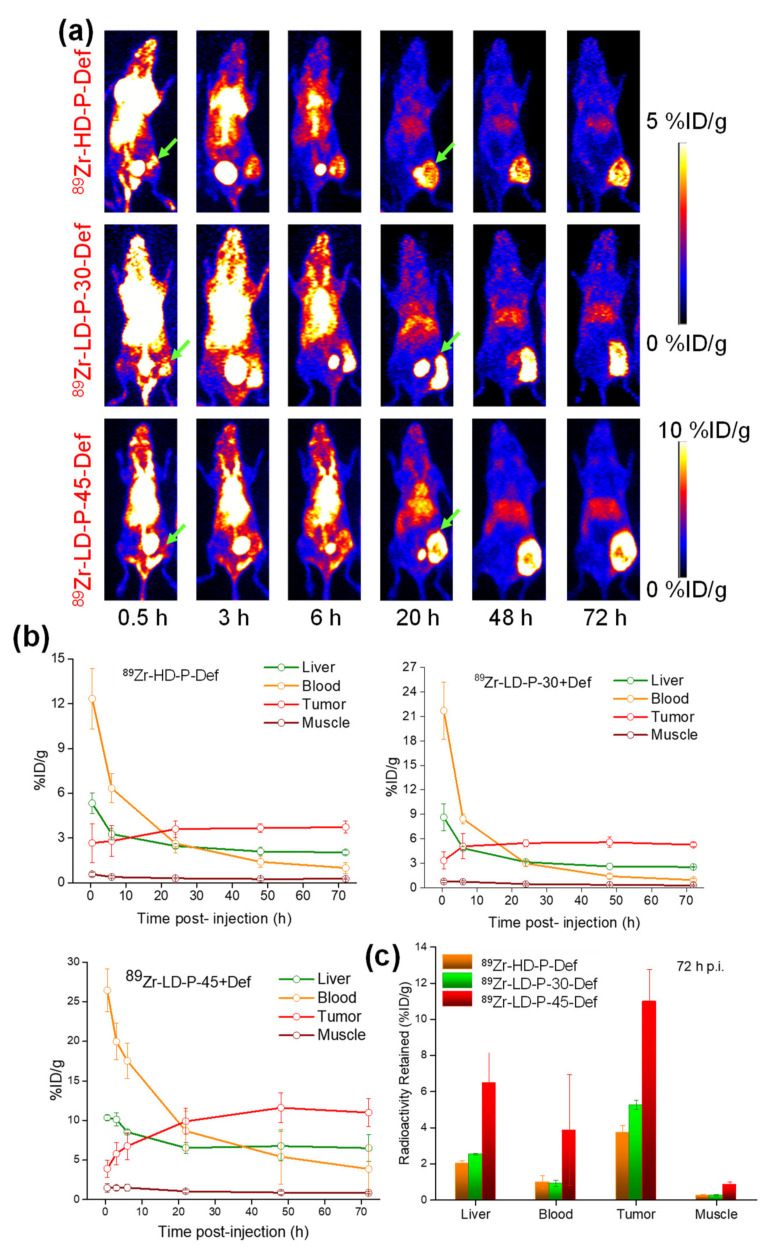
In vivo positron emission tomography (PET) imaging and biodistribution study: (**a**) serial maximum intensity projection (MIP) images, (**b**) time–activity curves, (**c**) and comparison of radioactivity retained in liver, blood, tumor and muscle of ^89^Zr-labeled linear pHPMAs; HD-P + Def (*M*_w_ = 27,800 g/mol, *Ð* = 1.74), ^89^Zr-LD-P-30 + Def (*M*_w_ = 33,300 g/mol, *Ð* = 1.06) and ^89^Zr-LD-P-45 + Def (*M*_w_ = 45,200 g/mol, *Ð* = 1.07). Reprinted with permission from [39]. Copyright (2017) The Royal Society of Chemistry.

The following more detailed study using fluorescently- or ^89^Zr-labeled polymer carriers differing in dispersity and also in *M*_w_ showed similar results [39]. The pHPMA characterized by low dispersity (*Ð* = 1.1) and *M*_w_ close to renal threshold (*M_w_* ≈45 kg/mol) prepared by RAFT polymerization exhibited the slowest blood clearance and the highest tumor accumulation, as was demonstrated by positron emission tomography (PET) on Figure 1.

Recently, also a thorough comparative study between polymer conjugates with THP bound by hydrazone bond differing in *M*_w_ (38,200 vs. 51,700 g/mol) and *Ð* (1.92 vs. 1.17) showed approximately two times higher accumulation in sarcoma S-180 tumors in the majority of time intervals for the low-dispersed conjugate with *M*_w_ about the renal threshold [40]. Importantly, a quite high amount of polymer was found in urine within the first hour in the case of the high-dispersed polymer conjugate. Consequently, prolonged blood circulation and higher accumulation resulted in higher antitumor activity. Nevertheless, although the trend was repeatedly documented in these studies, the increase was not always significant. It was found that both polymer conjugates, despite their different accumulation rate in tumors, exhibited a similar therapeutic effect on early-stage tumors (initial volume about 40 mm^3^), which have highly active angiogenesis and show better EPR effect. However, the efficacy of the low-dispersed conjugate was significantly higher than that of the high-dispersed conjugate during the treatment of well-developed S-180 tumors (initial volume about 150–250 mm^3^).

It can be concluded that pHPMAs characterized by quite low *Ð* and *M*_w_ near the limit of renal filtration is very promising carriers of imaging agents and/or drugs for highly efficient solid tumor treatment and diagnostics with minimal side effects.

Recently, linear pHPMAs have also been employed for the targeted delivery of anti-inflammatory drug dexamethasone [41,42]. Preferable accumulation of dexamethasone carrying nanomedicines within the inflamed tissue was detected, showing the potential of these nanomedicines to be passively accumulated within the inflammation tissue upon intravenous or intraperitoneal injection. Importantly, the intraperitoneal injection of these nanomedicines led to the highly elevated anti-inflammatory effect in the treatment of induced rheumatoid arthritis in mice or rats.

### 3.2. Biodegradable HMW Polymer Carriers

After it was recognized that pHPMAs accumulate in solid tumors due to the EPR effect in a molecular weight-dependent manner, various HMW biodegradable conjugates differing in the inner structure and biodegradability have been designed and synthesized. Four basic types of HMW nanomedicines have been designed and studied in detail, in, which diblock [43], multiblock [44,45,46,47], grafted [48], or star [49,50,51,52] structure was employed. All the HMW pHPMA constructs can be synthesized directly using modern controlled polymerization techniques [37,47] or by multiple-step synthesis. Star-shaped systems with high molecular weight could be synthesized via grafting-from approach utilizing the RAFT polymerization or via grafting to approach [52] employing the pre-prepared polymers for controlled grafting procedure.

As we discussed above in the Introduction, the effective extravasation of nanomedicines in solid tumors cannot be achieved above a certain limit. For example, star pHPMA above 600,000 g/mol, which corresponds to hydrodynamic size around 50 nm, exhibited markedly reduced accumulation in EL-4 lymphoma [14]. Moreover, also, there is a limit to renal filtration. While more flexible linear pHPMAs with *M*_w_ up to 70,000 g/mol can be excreted by the urine, more rigid star pHPMAs have a lower renal threshold of around 50,000 g/mol [17].

**Figure 2:**
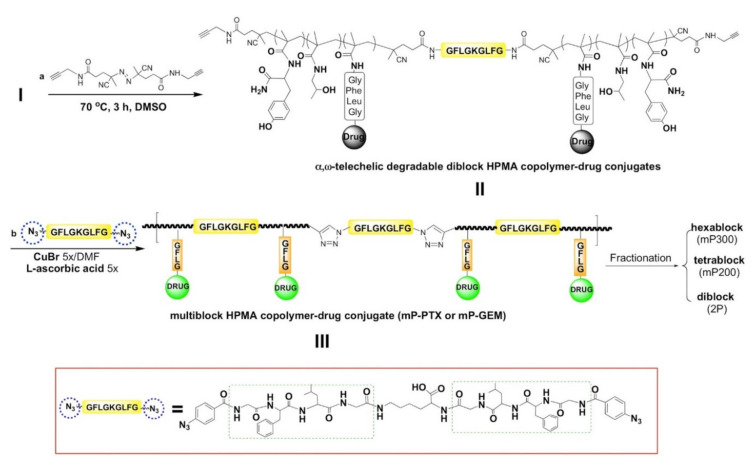
Synthesis of multiblock biodegradable *N-*(2-hydroxypropyl) methacrylamide (HPMA) copolymer (pHPMA)–drug conjugates. Reprinted with permission from [62]. Copyright (2017) American Chemical Society.

Biodegradable linear diblock or multiblock pHPMA drug carriers have been synthesized with the aim to create nanomedicines with prolonged blood circulation, and enhanced drug accumulation in solid tumors or inflamed tissues than that achieved previously by simple linear pHPMA. Disulfide spacers that are degraded reductively in the cytoplasm or GFLG spacers that are degraded enzymatically in lysosomes were positioned between polymer blocks, enabling intracellular polymer carrier degradation and the subsequent elimination of the resulting shorter degradation fragments. Importantly, the size of the polymer coil in solution controls the rate of polymer elimination by glomerular filtration rather than the polymer’s *M*_w_ per se, although the *M*_w_ is a convenient and easily calculated measure and is often used as a characteristic for the elimination limit of polymers. The resulting long-circulating carriers have been used to deliver potent drugs (Dox [53], THP [54], gemcitabine [55], paclitaxel [44], prostaglandin [53]) and also proved its suitability for combination therapy, thus delivering a combination of drugs [56,57,58]. Biodegradation of the diblock conjugates in solution modeling the intracellular condition resulted in the formation of polymeric degradation products with *M*_w_ values below the renal threshold [59]. Another HMW biodegradable multiblock carriers and conjugates have been synthesized using well-defined diblocks as a click reaction substrate. Diblock precursors have been synthesized via RAFT polymerization using a specific GFLG oligopeptide containing chain-transfer agent, [45,47,60] Figure 2. All these diblock and multiblock conjugates have been summarized and reviewed last year by Kopeček and Yang [61].

A new simplified approach for the synthesis of biodegradable diblock carriers was published recently. In the novel synthetic route, the diblock copolymers are directly formed from linear pHPMAs with TTc end groups during the removal of these groups with butylamine in water. The molar ratio of butylamine and the TTc group (20:1) was selected to reach a high reaction yield. The formed thiol groups on the polymer ends in situ reacted with each other to form a disulfide bond between polymer chains. The conversion reached its maximum after 1 h (from 80 up to 90% of diblock was formed) [63].

Grafted and branched polymer carriers received great attention in the 1990s and in the first decade of the current century. Today, they are out of the main interest of researchers and have been replaced by diblock or star-shaped structures due to controlled polymerization techniques. The results of grafted and branched pHPMA carriers have been partially summarized in a recent review [64].

The effect of size and 3D structure of pHPMA biomaterials on in vitro transport and in vivo organ accumulation was investigated thoroughly by Pearce et al. [65]. Through aqueous RAFT polymerization, they successfully produced a set of polymer materials spanning a size range from 5 to 60 nm, with the linear, hyperbranched, star, or self-assembling micellar structures and investigated the contribution of the structure, size and degradability on in vivo distribution by maintaining the same materials chemistry throughout. The results showed promising behavior of pHPMA biomaterials as stealth carriers both in vitro and in vivo. In vitro macrophage uptake studies demonstrated significantly different behaviors governed by surface zeta potential and size. The small hyperbranched structures were taken up by macrophages to a significantly lower degree than the larger hyperbranched and star constructs, which was in concordance with reduced mononuclear phagocytic system uptake and increased renal clearance in vivo. Hyperbranched and star carriers have been conjugated with anticancer drug Dox and showed improved efficacy over free drug in 2D and 3D cell culture models as well as in an aggressive orthotopic model of human triple-negative breast cancer in mice.

**Figure 3:**
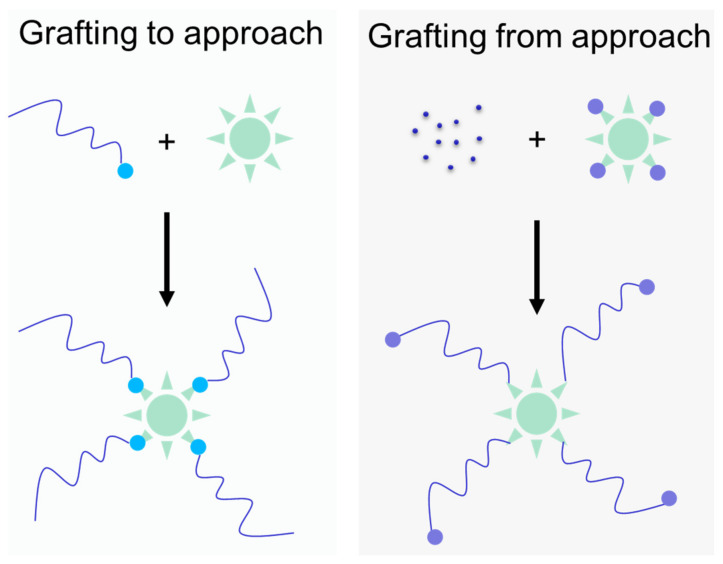
Schematic description of pHPMA-based star-like nanomedicine synthesis. The grafting-to approach is based on the covalent one-point attachment of semitelechelic polymer precursors (the light blue dot is reactive group on main chain end of polymer) onto the core (green star) containing functional groups. The grafting-from approach is employing the reversible addition–fragmentation transfer (RAFT) polymerization using the core containing several chain-transfer agents (violet dots) leading to the growth of the polymer chain from monomers (small violet dots) directly on the core.

The newest members of the HMW conjugate family are the star-like conjugates. Star-shaped carriers based on pHPMA have been recently summarized in two reviews [64,66]. The newest generation of star-shaped nanomedicines based on pHPMA was synthesized with the grafting to approach using a biodegradable hyperbranched polyester core based on 2,2-bis (hydroxymethyl)propionic acid (bisMPA), described first by Kostková et al. [67]. In general, also the grafting from approach could be employed along with the development of novel controlled RAFT polymerization techniques, Figure 3. The HMW star system containing hydrolytically degradable ester bonds on a bis-MPA core was constructed as a long-circulating polymer carrier, enabling prolonged drug circulation with highly enhanced accumulation in solid tumors. The time-dependent hydrolytic biodegradation of the HMW system in normoxic physiologic conditions in model buffers and human plasma ensures the safe elimination of polymer carriers from the body after fulfilling their function. Moreover, the pH-sensitive release of the active drug Dox in a hypoxic tumor microenvironment showed the stimuli-responsive behavior of the star polymer conjugates.

**Figure 4:**
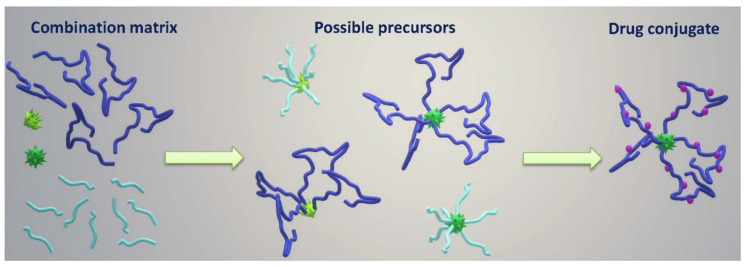
Schematic sketch of the formation of adjustable star-shaped nanomedicine based on semitelechelic pHPMAs and polyester-based core. Green stars are bisMPA cores, light and dark blue lines are polymers, violet dots represent drugs.

Recently, a whole library of star materials based on semitelechelic pHPMAs and bisMPA cores was described, and the biological behavior in mice tumor models was determined, Figure 4 [52]. The hydrodynamic diameter of the star copolymer biomaterials was tuned from 13 to 31 nm, with corresponding molar mass ranged from 87 to 720 kg/mol. Therefore, the star nanomedicines and their size could be adjusted to a given purpose by a proper selection of the bisMPA dendritic core type and generation and by considering the semitelechelic copolymer *M*_w_ and polymer-to-core molar ratio. The hydrolytic degradation was proved for both the star copolymers containing either dendron or dendrimer core, in aqueous buffers and plasma in vitro and in vivo using PET imaging. An excellent clearance from the body was shown in vivo for the dendron-based material, with more than 60% of biomaterial mass eliminated after 7 days. It has been shown unequivocally that the therapy by the biodegradable star conjugate with attached Dox strongly the tumor growth in mice and was fully curative in most of the treated animals at a dose corresponding approximately to one-fourth of the maximum tolerated dose (MTD). The newly developed biodegradable star nanomedicines showed superior efficacy and tumor accumulation over the first generation of star copolymers containing a non-degradable PAMAM core.

Indeed, the tumor spheroid penetration study showed identical penetration through spheroids of linear and star-like pHPMA and their constructs with pirarubicin. Nevertheless, the THP penetration after application of pHPMA conjugated THP was considerably deeper than for free THP, thus proving the benefits of polymer carriers, notwithstanding their inner structure [68]. Moreover, the cytotoxicity of THP conjugates against tumor cell spheroids was nearly the same as for free THP, whereas the 2D cell cytotoxicity of the pHPMA-conjugated drug is usually lower. Star-shape nanomedicines contain β- or γ-cyclodextrins as the biodegradable core have also been described recently [69]. Two synthetic approaches differing in the method of polymer grafting have been employed with the aim to obtain similar polymer carriers with different degradation rates.

### 3.3. Self-Assembled HMW Polymer Carriers

Another approach of how to prepare long-circulating HMW polymer carriers to consist of the utilization of self-assembled supramolecular structures, e.g., polymer micelles. Recently, micellar pHPMA nanomedicines with controlled degradation have been reviewed in detail [6,70]. Generally, amphiphilic copolymers self-assemble into supramolecular structures, usually termed as micelles, with a size exceeding the limit of the renal filtration. Moreover, polymer micelles disintegrate under their critical micellar concentration (CMC) into individual polymer chains, unimers, whose *M*_w_ should be under the limit of the renal threshold. The micelle-forming polymer carriers do not need to comprise any biodegradable linkages to enable their elimination from the body. It is a known fact that any shift in hydrophilicity of polymer carriers to a more hydrophobic nature could lead to undesired accumulation in the organism, often in the liver or other organs. Thus, there have been several attempts to disintegrate supramolecular structures after they deliver their cargo to the target tissue and facilitate their elimination from the body.

Typical structures of amphiphilic copolymers are block or graft copolymers. The hydrophobic blocks or molecules constitute the micelle core, which is surrounded by a hydrophilic shell formed by an HPMA homopolymer or copolymer, which should protect the micellar carrier from undesired interactions with proteins in blood and recognition by RES [6].

Amphiphilic block copolymers can be comprised of various diblock or triblock copolymers where the hydrophobic block consists of e.g., poly (laurylmethacrylate) [71], poly (ε-caprolactone) [72], poly (L-lactide) [73], poly (propyleneoxide) [74]. Moreover, the hydrophobic block can also be formed by pHPMA modified with valproate [75], or monolactate, dilactate, or benzoyl [76] on the hydroxyl group of HPMA. After hydrolyzes of these ester bonds, the hydrophilicity of the polymer carrier increased, and the micellar structure disintegrates. Similar behavior is also expected in the case of hydrolytically degradable polyesters core-containing amphiphilic copolymers mentioned above. Indeed, amphiphilic copolymers can also be designed as graft copolymers. Recently, semitelechelic pHPMA have been grafted to poly (ε-caprolactone) by azide-alkyne click reaction leading to the formation of amphiphilic copolymer self-assembling to micelles enabling both the physical entrapment of hydrophobic drugs, i.e., venetoclax, and covalent attachment via pH-sensitive hydrazone bond, i.e., Dox [77].

**Figure 5:**
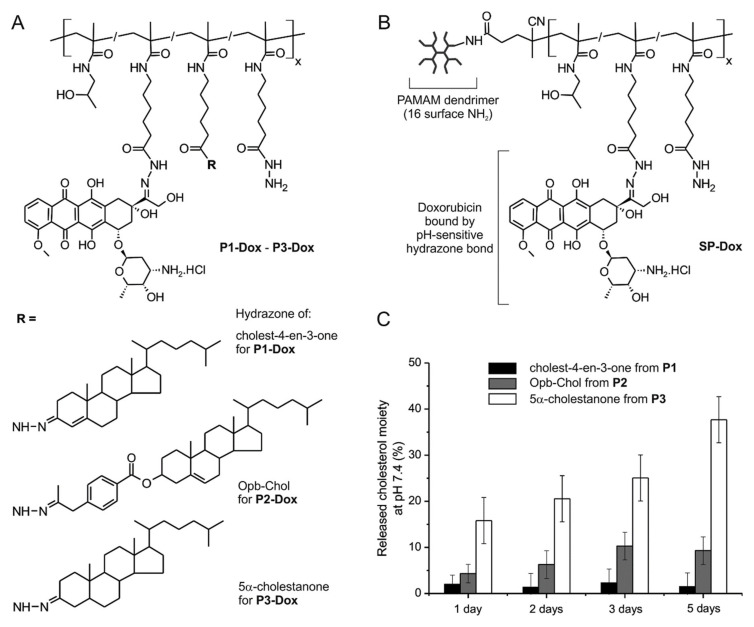
Schematic structure of amphiphilic pHPMA−doxorubicin (Dox) conjugates P1–P3 differing in the hydrophobic moiety (**A**) and star pHPMA−Dox conjugate (**B**). Release of cholesterol moieties from copolymers P1−P3 at pH 7.4 and 37 °C, mimicking the bloodstream environment (**C**). Reprinted with permission from [82]. Copyright© American Chemical Society (2018).

Another approach consists in the “decorating” of a hydrophilic polymer chain with rather small hydrophobic molecules. Oleyl, dodecyl, or various cholesterol-derived moieties have been attached to linear pHPMAs and used as carriers of Dox [78,79,80]. Alternatively, a hydrophobic moiety can be introduced into the hydrophilic polymer main chain end. For example, the presence of hexaleucine oligopeptide resulted in the formation of micelles [81]. Interestingly, hydrophobic moieties have been bound to pHPMA by means of a pH-sensitive hydrazone bond, enabling the tumor low pH-driven disintegration of supramolecular structure, Figure 5 [79,82]. In this case, the stability of the micellar structure at neutral pH strongly influenced the extent of their accumulation in solid tumors. The overall stability of micelles can be additionally improved by core crosslinking using, e.g., disulfide bridges [83] or hydrazone linkages [80], which can be further reduced by glutathione, or hydrolyzed in tumor cells, respectively. Another important feature for the successful utilization of amphiphilic polymer drug carriers in medicine is the absence of interaction with serum proteins, i.e., non-fouling behavior. Such proof was described for albumin and several other proteins and cholesterol-based pHPMA micelles [84,85,86].

Generally, drugs can be entrapped in the micellar core or bound by biodegradable bonds, which can enable precise control over drug release. Often, the covalent attachment of hydrophobic drugs switched the physicochemical properties of the conjugates to a more hydrophobic or amphiphilic nature according to the content and chemical structure of drugs. For example, the polymer coil of linear polymer conjugates with Dox bound by the hydrazone bond collapsed with the increasing content of the drug [87]. The second virial coefficient changed to negative at about 13 wt %, and at about 18 wt %, the formation of dynamic aggregates was observed. Such behavior was found even for different drugs, e.g., docetaxel, dexamethasone [11,56]. However, in the case of a much more hydrophobic drug, betulinic acid, the formation of micelles was determined [88]. Here, the micelles were disintegrated after the drug derivative release in an acidic condition of tumor cells and thus facilitated polymer carrier elimination.

A specific part of micelle-forming polymer carriers represents thermoresponsive copolymers that are characterized by low critical solution temperature. They form micelles above a certain temperature, while they are fully soluble as polymer coils at room temperature, which is important for the simple and define preparation of micellar samples. Block or statistical copolymers of HPMA and *N*-isopropylacrylamide (NIPAM) [89], 2-(2-methoxyethoxy) ethyl methacrylate (DEGMA) [90,91], or HPMA-dilactate [92] have been synthesized and transition temperature set by tuning of monomer ratio.

## 4. Augmentation of the Passive Accumulation in Solid Tumors

Current clinical results of conventional chemotherapy are still not appropriate even they are used in clinical practice for more than 60 years [93]. The major issue in the insufficient anticancer efficacy is driven by the lack of tumor selectivity of such anticancer drugs. Thus, the development of selective tumor-targeted drug systems, i.e., nanomedicines, is an urgent need in current anticancer therapy. Within the last decades, molecular drugs have attracted serious attention, as they target important particular molecules, growth factors, and/or specific oncogenes highly expressed by tumor cells. Their preclinical results were highly positive, showing their serious potential to treat tumor cells of various origins. Nevertheless, recently described results of the clinical investigation have not fully confirmed the positive expectations. These investigations showed unsatisfactory results in the efficacy of molecular target drugs, especially for solid tumors [94]. There are several pieces of evidence that the intrinsic heterogeneity of tumors and several mutations in individual patients may lead to the failure of these treatments [95]. It is believed that the intratumor heterogeneity, associated with heterogeneous protein function, can cause and foster tumor adaptation and therapeutic failure through a Darwinian selection of tumor cells.

Moreover, even nanomedicines showing excellent efficacy in mice models when intravenously injected do not effectively reach the tumors due to the biological barriers in the body [96]. Importantly, the use of nanomedicines in humans often resulted in a lack of overall patient response and survival [97]. The PEGylated liposomal Dox nanoformulations (Doxil^®^) generally reached safety improvements, but not an increase in efficacy compared to the standard therapies [98]. Recently, considerable effort has been expended to develop advanced nanomedicines alternative to the approved liposomal formulations; unfortunately, their clinical translation has been frequently depleted by various technical, cost, and efficacy-related issues. Thus, skepticism about the use of nanomedicines increased in the scientific community in recent years [99].

Extensive angiogenesis is the key factor in tumorigenesis of early growth stages of solid tumors, thus enabling accelerated tumor growth as the cancer cells are fully supplied by nutrients and oxygen. As a consequence, the early-staged solid tumors are often endowed with higher vascular density compared with normal healthy tissues. Indeed, for large-size tumors, more precisely in late-stages, the vascular blood flow is, on the contrary, seriously obstructed. In that case, the needs of tumor cells are not fulfilled, as the vascular oxygen supply and nutrients are not sufficiently delivered, and tumor tissues become strongly hypoxic, the tumor cells are dying, and tumors become avascular [100].

Nevertheless, most clinical tumors are large and advanced or late-stage tumors, and their structure is known for the necrotic and avascular areas that lead to an insufficient EPR effect [101]. Recently, it was found that tumor tissue coagulation or thrombogenicity was highly enhanced as tumors grew up [102,103], which lead to the occlusion and blocking of tumor blood vessels and consequently to a poor EPR effect, highly depleting the success of cancer chemotherapy in advanced cancer. Moreover, tumor interstitial fluid pressure (IFP) has become an important barrier to efficient drug delivery via the EPR effect [104]. Most solid tumors are connected with increased IFP, which is most probably linked to the osmotic pressure of the extravasated fluid and the dysfunctional lymphatic system of tumor tissues. Importantly, the rapid growth of tumors reporting a short doubling time of 24 h to 1 week will, in addition, enhance the physical and mechanical pressure that can be even summed up with osmotic pressure, which is caused by increases in tumor mass [93]. In summary, advanced large tumors are frequently heterogeneous containing regions of defective vasculature and highly restricted blood flow, which finally deplete the EPR effect and linked drug delivery to tumors.

**Figure 6:**
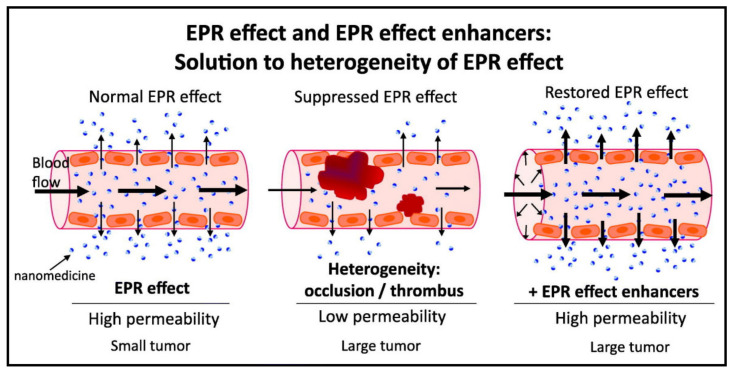
Schematic description of the enhanced and permeability retention (EPR) effect and application of EPR effect enhancers for the solution of heterogenicity of tumor tissue. Reprinted with permission from [101]. Copyright (2020) Elsevier B.V. (Amsterdam, Netherlands).

Recently, a novel approach based on the augmentation of the EPR effect was described [101,105]. Various vascular mediators, the nitric oxide (NO) generators nitroglycerin (NG) [106], hydroxyurea [107], and L-arginine [105], have been studied as potential enhancers of the EPR effect in order to improve the therapeutic effect of nanomedicines (Figure 6). It was described that all the studied compounds are able to generate the NO with relatively high selectivity in solid tumors [105]. The augmentation of therapeutic effect via the EPR effect enhancement was studied in detail using pHPMA nanomedicines carrying cytostatic drug THP, or photodynamic therapy (PDT) nanoprobes pyropheophorbide-a, or zinc protoporphyrin. Interestingly, the NO-donor–base augmentation significantly increased, almost twice, the tumor accumulation of nanomedicines and nanoprobes in various solid tumor models. As a consequence, the antitumor effects, either cytostatic or PDT-based, were also markedly improved, showing the potential for further clinical application. Indeed, in a murine autochthonous colon tumor, NO donors markedly enhanced the therapeutic effects of THP bearing pHPMA even after one single administration, and the therapy outcome was comparable with those achieved with three weekly nanomedicines treatments. Moreover, a similar positive effect of the NO donors was described in the compassionate use in human trials. Nitroglycerine was used to increase the efficacy of the THP bearing polymer conjugates in a patient with stage IV prostate cancer [108]. The augmentation of the EPR effect, in this case, led to the enhanced efficacy proving even the remission of the lung and bone metastasis.

Similarly, carbon monoxide (CO) was utilized as a potential enhancer of the EPR effect. Recently, Fang et al. employed two CO generating agents, either extrinsic CO donor micelle containing tricarbonyldichlororuthenium (II) dimer or endogenous CO donor using PEGylated hemin inducing heme oxygenase-1 [109]. It was proved that the agents induced the generation of CO selectively in solid tumors, thus enhanced the EPR effect leading to a two- to three-fold increased tumor accumulation of used nanomedicines. Importantly, the CO enhancers worked similarly for the pHPMA nanomedicines containing THP as well as for the pHPMA nanoprobe with pyropheophorbide-a. The application of CO generators altogether with anticancer nanomedicines resulted in a significant increase of efficacy in various transplanted solid tumor models.

**Figure 7:**
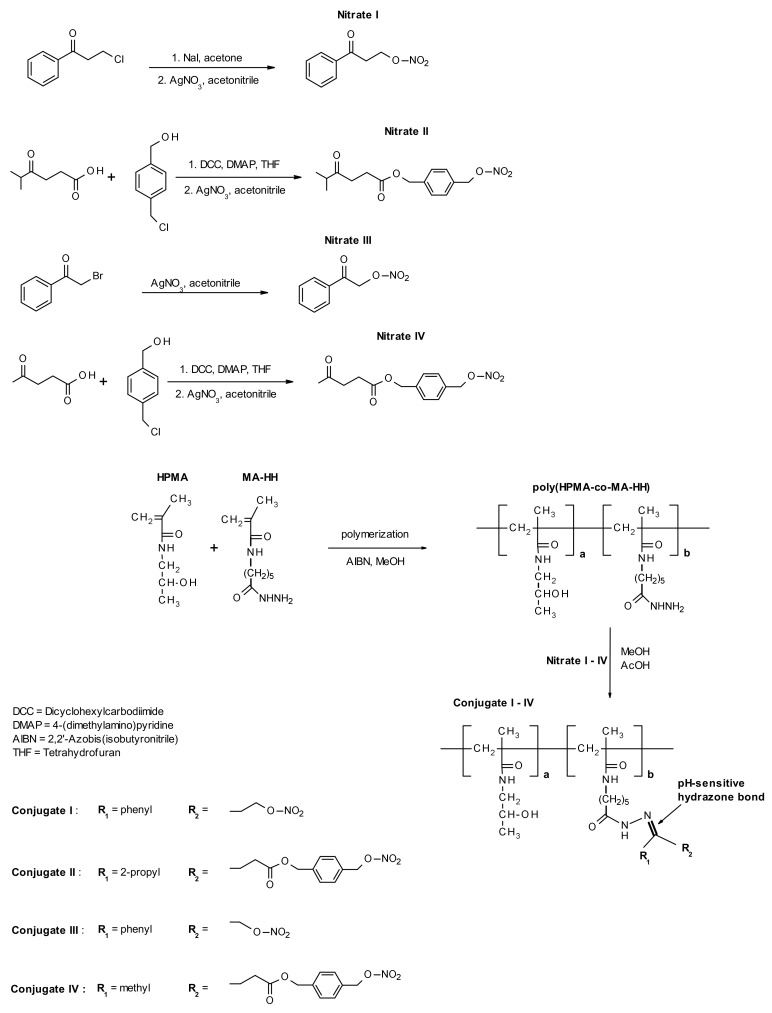
Synthesis of polymer-bound nitric oxide (NO) donors. Reprinted with permission from [110]. Copyright (2018) Elsevier B.V. (Amsterdam, Netherlands).

As mentioned above, the utilization of low-molecular-weight NO and CO donors leads to the enhancement of nanomedicine accumulation and efficacy in the treatment of various solid tumor models. Nevertheless, the use of small NO and CO donors could also cause vasodilatation in healthy organs leading in combination with nanomedicines to some obstructions. Thus, another approach based on the binding of the organic nitrate precursor of NO to a water-soluble pHPMAs was published [110], Figure 7. Four different pHPMA-bound NO donors differing in structure and hydrolytic stability have been investigated. These polymer-bound NO donors have been able to overcome some drawbacks related to low-molecular-weight NO-releasing compounds, namely systemic toxicity, lack of site-specificity, and fast blood clearance.

A significant increase in the EPR effect was found for pHPMA-Dox conjugate in a murine lymphoma model. The augmentation of the EPR effect enhanced the therapeutic outcome of Dox-containing nanomedicines, but not of free Dox. Similarly, the study using an *S*-nitrosated human serum albumin dimer was recently published, showing the synergistic effect when used as a pretreatment agent in albumin-bound paclitaxel nanoparticle (Abraxane^®^) therapy carried on various tumor models [111,112]. Interestingly, in the C26 murine colon cancer, the NO-generating dimer enhanced tumor selectivity of paclitaxel containing nanoparticle and attenuated myelosuppression. Augmentation of the tumor growth inhibition during the treatment by paclitaxel bearing nanoparticles was also seen in the low vascular permeable B16 murine melanoma model. In summary, the tumor-site localized augmentation of the EPR effect via the polymer-bound NO delivery system is recognized as a highly promising strategy to a highly potentiate nanomedicines-based tumor therapy without increasing systemic toxicity. The proper selection of delivering vectors altogether with the proper NO release profile should be investigated for further development.

## 5. Active Targeting Versus Passive Accumulation of pHPMA Nanomedicines

Generally, targeted nanomedicines have several advantages, which lays mainly in the protection of healthy cells during the treatment, serious reduction of the side adverse effects, and overcoming various biological barriers making the cancer cells highly resistant to the treatment [6]. Highly specific characteristics of the tumor tissue in general and of its microenvironment made it possible to design nanomedicines that are able to deliver biologically active molecules, e.g., drugs, to tumors [113]. Conventional anticancer drugs show inappropriate pharmacokinetics and are localized within the body nonspecifically. Thus, it is impossible to solely reach the target tumor tissue, and their use is associated with serious side effects. The development of nanomedicines enabled more favorable pharmacokinetics and enhanced tumor accumulation. It has been generally accepted that nanomedicines can reach solid tumors through the leaky vasculature utilizing the above-mentioned EPR effect. Nevertheless, specific targeting of the tumor tissue is still a remaining challenge for researchers around the world, especially in the case of poorly vascularized and dispersed tumors. The interest has been focused on nanomedicines bearing specific molecules enabling to mediate the active targeting by selective interaction to the receptors overexpressed on the cancer cells or tumor endothelium. Recently, a huge number of reviews was published discussing the possibility and pros and cons of passive nanomedicine accumulation and active targeting [114,115].

Already in the 1990s, Seymour et al. described the influence of *M*_w_ of pHPMA on passive accumulation in tumors, namely sarcoma 180 or B16F10 melanoma models [116]. From this time, an enormous number of reports describing the dependence of passive accumulation of polymers onto their structure, *M*_w_ and size was published [5,117,118]. The tumor-selective accumulation was improved either by the synthesis of more complex structures, e.g., grafted, multiblock, or star polymers, or by the controlled self-assembly of the amphiphilic HPMA copolymers [66,70], or by the utilization of various EPR effect enhancers as we discussed in previous chapters.

On the other hand, many attempts have been made to design and synthesize actively targeted pHPMA nanomedicines. Various targeting ligands, e.g., monoclonal antibodies, immunoglobulins, peptides, lectins, saccharides, have been employed and studied in detail [6]. In general, the two targeting approaches have been employed, i.e., direct targeting to cell receptors overexpressed on the tumor cell or tumor endothelium as the final destination of the targeting ligand. Generally, the targeting efficacy was found significantly higher for the monoclonal antibodies in contrast to smaller molecular weight targeting ligands. Even in the case of multiple presentations of oligopeptides originated from the active site of antibodies, it is not able to reach the same affinity to their target ligands [119]. Recently, the targeting efficacy of antibody-targeted pHPMA nanomedicines was reviewed in detail [120].

Importantly, H. Maeda recently analyzed repeated failures in cancer therapy for solid tumors [93]. Regardless of the huge financial support of bullet-like therapies targeting site-specific cancers, i.e., molecular drugs for the depletion of specific enzymes such as kinases or inhibitors of growth factor receptors, the therapeutic results are unsatisfactory and disappointing. The main scientific reasons leading to the malfunction of the mentioned drug development approaches should be linked to the infinite number of genetic mutations in a chaotic molecular environment of solid tumor tissue. It was found, the outcome failure rates of approximately 90% on current therapeutic approaches for solid tumors are estimated. Partial success was achieved with drugs such as Gleevec or few other molecules that are used for treating patients with hematopoietic cancers and soft tissue or seminoma. Similar to the new molecular therapies, the active targeting in the case of solid tumors reach the limitation of a huge number of genetic mutations in the tumor environment, which strongly suppresses the overall targeting ability of solid tumors. Nevertheless, the antibody-targeted pHPMA nanomedicines reached significant benefits in the treatment efficacy in the case of various hematological malignancies studies in vivo. Various lymphomas [121,122] and leukemias [123,124] have been efficiently eradicated by the antibody-targeted pHPMA conjugates, showing the potential of the active targeting in the case of hematological malignancies, which are known for low genetic mutations in contrast to other solid tumors [93].

Indeed, the efficacy of the active targeting was also thoroughly studied in a time-dependent manner to prove the potential benefit of the active targeting. Either epidermal growth factor (EGF)-based [125] or tumor endothelium-based targeting [119] have been employed to study the time dependence of the active targeting. In both cases, it was observed that the active targeting is worth being effective in short times up to the 1 or 4 h, respectively. After that time, the passive accumulation of nanomedicines with similar hydrodynamic sizes reach the same accumulation as the actively targeted polymer conjugates. Specifically, in mice bearing both highly leaky CT26 and poorly leaky BxPC3 tumors, it was observed that tumor vascular endothelium could be targeted effectively, showing the rapid and efficient early binding to tumor blood vessels [125]. Nevertheless, over a short period of time, the passive targeting based on the EPR-driven accumulation highly prevailed, leading to a higher overall accumulation. Similarly, the EGF-targeting to FaDu head and neck carcinoma in mice showed a short time effective prevalence of the active targeting showing the rapid accumulation in tumors within 15 min. Nevertheless, after 4 h, the nontargeted star-like nanosystems reached the same accumulation of similarly sized antibody-targeted conjugates [119].

In summary, the active targeting seems to be suitable for the design of highly effective nanomedicines, especially against the hematological malignancies, where in the last decade, several antibody–drug conjugates have been approved for clinical use [120]. In the case of solid tumors of other origins, it seems that the passive targeting based on the tumor microenvironment abnormalities is more favorable and should be taken into consideration more frequently.

## 6. Future Prospects

Within the last three decades, a serious number of structures of pHPMA prodrugs have been designed, synthesized and their properties have been described. Even though most of them showed highly promising therapeutic activity or imaging properties in animal models during preclinical development, only a few of them came to any clinical evaluation. Unfortunately, none of them have been approved so far for clinics and marketed yet.

Nevertheless, for future prospects, the application of novel controlled polymerization techniques and advanced synthetic routes, including click chemistry and oriented binding, should enlarge the potential of the wider exploitation of pHPMA-prodrug-based nanomedicines, as shown recently in compassionate clinical use [108]. Employment of tailored tumor-, inflammation- or bacteria-linked stimuli-sensitive spacers should enlarge the importance of the controlled drug release. Similarly, controlled biodegradability of novel pHPMA structures should lead to the next-generation of pHPMA nanomedicines with higher clinical potential. Thus, to sum up, the design of tailor-made pHPMA nanomedicines with increased specificity of tissue- or cell-specific drug delivery is a promising step in terms of future applicability of these prodrugs.

It was shown that the efficacy of passively targeted nanomedicines could be highly improved by the controlled application of various EPR-effect enhanced, both the low-molecular-weight compounds and polymer-based enhancers. Most probably, the application combining the augmentation of the EPR effect with tailored nanomedicines will improve the therapeutic efficacy of such polymer–drug conjugates and thus even their clinical usefulness. Last, but not least, the strong potential is envisioned also in controlled drug delivery for the treatment of specific inflammatory diseases, i.e., site-specific rheumatic musculoskeletal diseases or bacterial infections.

Nevertheless, recently a study showing that the interendothelial gaps in the tumor endothelium are not responsible for the transport of nanoparticles into solid tumors was published [126]. Importantly, the authors found that up to 97% of nanoparticles are entering tumors using an active process through endothelial cells. These results could open a new paradigm for developing cancer nanomedicines and could suggest novel approaches using the understanding of these active pathways to unlock strategies to enhance tumor accumulation.
